# Familiar size affects perception differently in virtual reality and the real world

**DOI:** 10.1098/rstb.2021.0464

**Published:** 2023-01-30

**Authors:** Anna M. Rzepka, Kieran J. Hussey, Margaret V. Maltz, Karsten Babin, Laurie M. Wilcox, Jody C. Culham

**Affiliations:** ^1^ Neuroscience Program, University of Western Ontario, Western Interdisciplinary Research Building, London, ON, Canada N6A 3K7; ^2^ Department of Psychology, University of Western Ontario, Western Interdisciplinary Research Building, London, ON, Canada N6A 3K7; ^3^ Department of Psychology, York University, Toronto, ON, Canada M3J 1P3

**Keywords:** virtual reality, familiar size effect, size perception, distance perception, head-mounted display, binocular vision

## Abstract

The promise of virtual reality (VR) as a tool for perceptual and cognitive research rests on the assumption that perception in virtual environments generalizes to the real world. Here, we conducted two experiments to compare size and distance perception between VR and physical reality (Maltz *et al*. 2021 *J. Vis.* **21**, 1–18). In experiment 1, we used VR to present dice and Rubik's cubes at their typical sizes or reversed sizes at distances that maintained a constant visual angle. After viewing the stimuli binocularly (to provide vergence and disparity information) or monocularly, participants manually estimated perceived size and distance. Unlike physical reality, where participants relied less on familiar size and more on presented size during binocular versus monocular viewing, in VR participants relied heavily on familiar size regardless of the availability of binocular cues. In experiment 2, we demonstrated that the effects in VR generalized to other stimuli and to a higher quality VR headset. These results suggest that the use of binocular cues and familiar size differs substantially between virtual and physical reality. A deeper understanding of perceptual differences is necessary before assuming that research outcomes from VR will generalize to the real world.

This article is part of a discussion meeting issue ‘New approaches to 3D vision’.

## 1. Introduction

The advent of affordable consumer-grade virtual reality (VR) has opened new possibilities for research in cognitive neuroscience and its applications in the real world. The potential for VR in research is particularly promising considering that a growing body of evidence suggesting that traditional laboratory settings may be inadequate proxies for reality [1]. In turn, VR may provide a useful balance between naturalism and experimental control, without the practical limitations of laboratory settings [2]. Moreover, virtual environments can be manipulated in ways that are not possible in a laboratory setting [2,3], opening new avenues for human perception and cognition research [4]. In such use cases, it is important that we understand the nature and scope of distortions that could impact the accuracy and precision of interactions specific to virtual environments.

Research and real-world applications of VR rest on the assumption that perception in VR is generalizable to perception in the real world. However, many head-mounted displays (HMDs) have constraints on realism, including narrow fields of view, graphical limitations, rendering distortions and obtrusive hardware. Although these limitations are headset-dependent, VR is limited more broadly by a lack of haptic cues [5] and the presence of the vergence-accommodation conflict (VAC; [6,7]). In addition, software for rendering VR content (e.g. Unity) was developed largely for gaming, which prioritizes rendering speed over accuracy. The limitations of VR technology raise concerns about whether VR enables sufficiently veridical perception for research and real-world applications.

One domain where perception has been found to differ between VR and reality is spatial perception. Both size and distance are perceived less accurately in VR than reality, with systematic underestimations of size and distance typically observed (e.g. [8–14]). The cause of geometric distortions in VR is unclear and cannot be fully attributed to reduced field of view [15–17], the physical weight of HMDs [18,19], or graphics quality [20]. Experiments in Cave Automatic Virtual Environments have demonstrated that size perception depends upon multiple sources of information. Most relevant to our studies, there is evidence that size constancy—the ability to perceive an object as being the same size as distance changes—improves as more objects are present in the virtual scene [20,21] and when stereopsis is available [21], but is relatively unaffected by accommodation [20] and motion parallax [21].

Critically, many studies on size and distance perception in VR have employed neutral stimuli (e.g. neutral spheres or cubes; [9,10]), as opposed to recognizable objects. Yet, size and distance perception is guided not only by bottom-up information such as vergence, but also by top-down cognitive processes. Arguably the most important cognitive factor impacting size and distance perception is familiar size [22]. Knowledge of the familiar size is acquired through experiences with the typical (familiar) spatial extent of objects and can be used to infer size, and in turn, distance of objects [23]. The influence of object identity on size and distance perception is known as the familiar size effect (FSE).

Although the importance of familiar size for perception has been debated [24,25], there is considerable evidence that learned object properties, such as familiar size, impact visual perception substantially. We previously found that familiar size significantly affected perception of real, tangible 3D objects, even when binocular cues to distance were available [26]. In that study, participants estimated the size of and distance to real Rubik's cubes and dice at both their familiar and unfamiliar sizes presented within 1 m from the participant. We found that when objects were presented at unfamiliar sizes, perception of size and distance were skewed towards the familiar size of the object. However, the FSE was strongest when oculomotor cues to distance (and thereby inferred size) were eliminated or minimized (by monocular viewing through a pinhole to remove vergence and accommodation). These results support the view that we rely more on cognitive factors when other visual depth cues are limited.

The objective of the current study was to compare the relative impact of familiar size and binocular vision on size and distance perception between the real world (based on data from Maltz *et al*. [26]) and VR (based on new data). There is a large body of literature on the study of distance, size and depth perception; the novel contribution of our study is the investigation of the relative contributions of familiar size and presented size on perceived size and distance in both VR and the real world. Furthermore, the direct contrast between real and virtual stimuli gives insight into the contribution of vergence and accommodation, and the conflict between them, to space perception. Notably, there is debate as to the degree to which vergence contributes to distance perception, particularly when it is in decoupled from other sources of depth information as in VR displays [27–30].

In experiment 1, we replicated our previous real-world study [26] in VR to directly compare size and distance perception of familiar objects between reality and VR under binocular and monocular viewing. Although past research has found that participants are sensitive to familiar size in VR environments (e.g. [31–33]), the novel aspect of our study is the direct contrast between real and virtual stimuli. As a starting point, we hypothesize that if perceived size is processed in VR as it is in the real world, then size and distance perception should be similar between the two environments. Specifically, in that case, familiar size should have a stronger effect on size and distance perception under monocular viewing than binocular viewing. Alternatively, if the limitations of VR (such as the VAC) result in a down-weighting of binocular cues to distance then the relative contribution of familiar size versus binocular vision would be expected to show a corresponding change in VR, compared to the real world. In experiment 2, we assessed how the results from experiment 1 would generalize to a more diverse set of virtual stimuli when viewed with a higher quality HMD (improved resolution, larger field of view and more accurate interpupillary distance (IPD)). We hypothesized that perception would be comparable in VR regardless of the specific stimuli tested and the quality of the VR display.

## 2. Experiment 1

### (a) Methods

#### (i) Participants

Twenty-two participants (age range 18–25 years) were recruited from the undergraduate psychology research participant pool at Western University. Inclusion criteria consisted of right-handedness, normal or corrected-to-normal visual acuity, no neurological conditions, no history of strabismus, familiarity with both dice and Rubik's cubes and normal depth perception (thresholds of 50 s of arc on the Randot Stereotest^TM^ or better). Owing to the potential risks of nausea associated with HMD use, participants who self-reported a history of motion sickness were excluded. All participants were naïve as to the purpose of the experiment. At the start of the session, participants signed informed consent forms that were approved by the Non-Medical Research Ethics Board of the University of Western Ontario (protocol ID 105313), in accordance with the 1964 Declaration of Helsinki. Participants received course credit for their participation. Data from the 22 participants in this study in VR were compared with data from the 32 participants in physical reality in the Maltz *et al*. [26] study, with no overlap in participants. Our sample size was smaller than that of Maltz *et al*. because that study demonstrated large and highly significant effects (see tables 1 and 2 of Maltz *et al*. [26]).

#### (ii) Apparatus and stimuli

**Figure 1.**
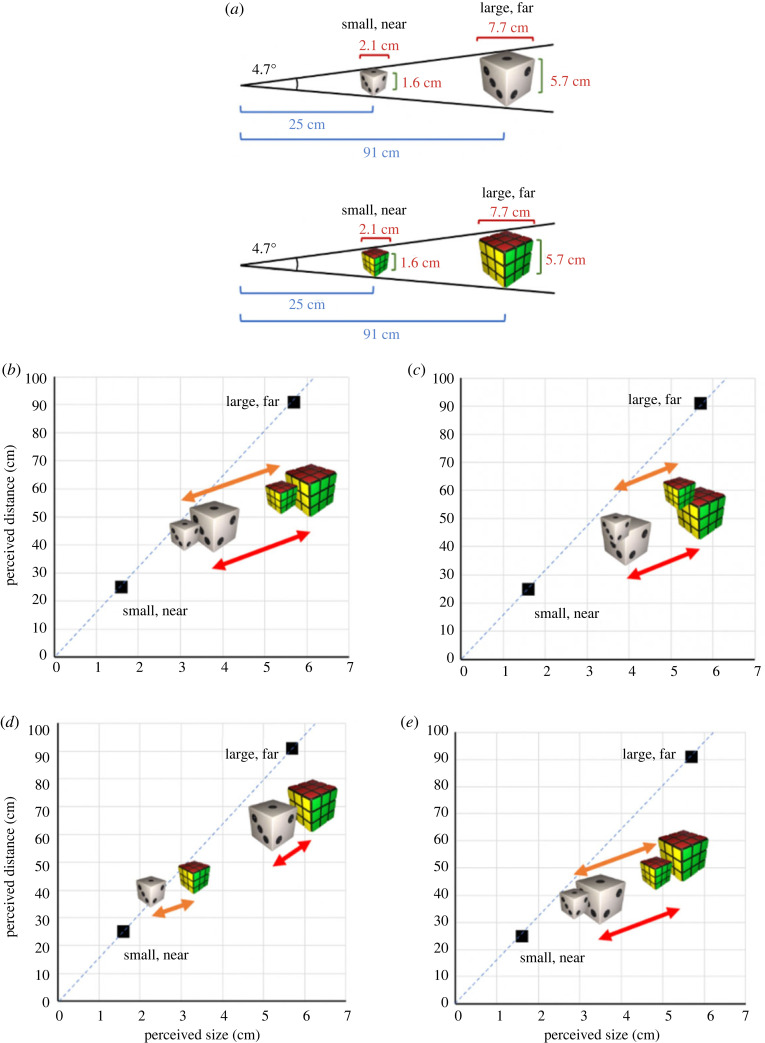
Comparisons of size and distance perception for virtual objects (experiment 1) and real objects ((*d*,*e*) adapted from Maltz *et al*. [26]). (*a*) Rubik's cubes and dice were presented as small/near or large/far, such that they subtended a constant visual angle of 4.7 degrees. Side length, longest axis length and distance from participant are indicated. Qualitative comparisons of presented size and distance to perceived size and distance in (*b*) VR binocular viewing, (*c*) VR monocular viewing, (*d*) real binocular viewing, and (*e*) real monocular viewing. The *x*-axis of each figure shows the perceived size of each object, and the *y*-axis shows the perceived distance of the object away from the viewer. The black squares indicate the presented size and distance of small, near and large, far objects; the dashed line represents combinations of size and distance that result in a constant retinal angle of 4.7°. The icons of Rubik's cubes and dice represent the average of the perceived size and distance of the pictured objects. The orange and red arrows represent the magnitude of the FSE (difference in perception for Rubik's cube versus die with the same presented size/distance) for small, near objects and large, far objects, respectively. Notably, FSEs were smaller for real objects under binocular viewing than in any of the other three conditions. (Online version in colour.)

Using an Oculus Rift CV-1 HMD, participants viewed virtual Rubik's cubes and dice rendered at their expected (congruent) sizes (5.7 cm Rubik's cube, 1.6 cm die) and at each other's (incongruent) sizes (1.6 cm Rubik's cube, 5.7 cm die). When presented, large stimuli were 7.7 cm at their widest axis and small stimuli were 2.1 at their widest axis. Each stimulus size was presented at a distance that subtended a constant retinal angle of 4.7° at the object's widest point (figure 1*a*). That is, small stimuli were presented at 25 cm from the participant (small/near condition), and large stimuli were presented at 91 cm from the participant (large/far condition). Distances refer to the point of the cube closest to the participant. We will use the terms ‘presented size’ and ‘presented distance’ to refer to the spatial dimensions simulated by rendering in VR or physically present in reality. Unlike the real-object study (Maltz *et al*. [26]), we did not present conditions with extreme visual angles owing to limitations of the VR display. Specifically, the resolution of the Oculus Rift CV-1 (9.8 pixels degree^−1^) was too coarse to accurately render the small/far object, and the large/near objects were uncomfortable to view owing to lens distortions outside the central 10 degrees of view. Consistent with the real-object study, all stimuli were presented against a black background, and anecdotally appeared to ‘float in a dark void’. Except where otherwise indicated, methods were consistent with those specified in greater detail in Maltz *et al*. [26].

All objects were constructed and presented in Unity. To create life-like stimuli, photographs were taken of a real die and Rubik's cube. Photographs were used to create a set of diffuse map textures that were used to define the surfaces of each object's main colours. The diffuse textures also provided the necessary information to help accurately light the objects within Unity. To form the shape of the objects, three mesh planes were orientated and snapped together to form a three-sided cube. The diffuse map textures were applied to the appropriate sides of the cube. Stimuli were rendered using Unity software at the required sizes and distances. Distances in Unity were validated by placing trackers at the desired distances in the real world and ensuring the readout was correct. During rendering, lighting was controlled to minimize any shading cues.

As in Maltz *et al*.'s study [26], objects were presented in both binocular and monocular pinhole conditions. The monocular pinhole was created by placing glasses with a single pinhole (0.7 mm in diameter) over the dominant eye (between the eye and the HMD screen). Because VR displays have fixed accommodation, the pinhole was not necessary to remove potential cues to depth from accommodation in VR; nevertheless, in experiment 1, we retained the pinhole to ensure that viewing conditions (including diffraction through the pinhole) were consistent with the real-object experiment.

Participants manually indicated the perceived size and distance of the presented objects. Manual estimation is a well-established common measure of perception for size and distance (e.g. [34]). Manual estimates have been shown to evoke almost identical results for depth perception as compared other perceptual tasks in both experienced and inexperienced observers [35]. That said, caveats apply; in particular, manual estimates must fall within the range of the effector span [36].

In manual estimation, our participants used the distance between their right index finger and right thumb to match the perceived size. When estimating distance, they spread their arms apart such that the distance between the two extended index fingertips matched the (orthogonal) perceived distance to the target. Size and distance measurements were quantified with a motion capture system (using two OptoTrak 3020 units, Northern Digital Inc.) to record the positions of three infrared-emitting diodes (IREDs) taped to the tip of the left index finger, right index finger and right thumb of each participant.

Participants wore the HMD and were seated in the same experimental space as the real-object study (i.e. in front of the original table and viewing tunnel; Maltz *et al*. [26]). The participant's chin was placed in a chinrest that was securely mounted to a table and adjusted to ensure participant comfort. The chinrest served to secure the head in a stationary position and limit motion parallax as a depth cue. A trial initiation button was located 25 cm to the right of the chinrest. A foot pedal was located under the table, and its activation triggered recording of finger coordinates by the OptoTrak sensors.

#### (iii) Procedure

Participants first completed The Edinburgh Handedness Inventory (Oldfield [37]), the Randot Stereotest, and an eye dominance assessment. Participants were also asked to draw the actual physical size of a Rubik's cube and die from memory to ensure familiarity with typical object sizes. IREDs were then taped to participants' fingers and verbal instructions were given. Participants first completed eight calibration trials with wooden calibration objects to assess IRED positioning relative to grip aperture. At this point, the HMD was placed on the head, the lights in the experimental room were turned off, and two practice trials with neutral wooden blocks were conducted.

Stimulus presentation order was blocked by viewing condition (binocular or monocular pinhole viewing), with the order of presentation counterbalanced between participants. Within each block, all trials of one size/distance combination preceded the other (consistent with Maltz *et al*. [26] for comparison with a related neuroimaging experiment that required this design). Within size/distance groupings, object identity alternated between the Rubik's cube and die (in counterbalanced order). Each combination of viewing condition, size/distance and object identity was presented four times.

Participants initiated a trial by pressing a button with their right hand, after which they heard an automated voice instructing them to either estimate size or distance first (in counterbalanced order). The stimulus then appeared and remained visible as long as the button was held. Participants were instructed to hold the stimulus presentation button only for as long as needed to gain a first impression of the object. Upon release, the stimulus disappeared and participants had 10 s to perform size and distance estimations. Once participants were satisfied that their manual estimation matched their perception, they pressed the foot pedal to register their estimate. A high-pitched tone indicated the termination of the response period, at which point the participant could initiate the next trial. If participants did not complete the task within the 10 s time limit, they heard a different signal and repeated the trial immediately. Additionally, if relevant IREDs were not tracked during the pedal press (i.e. owing to hand position obscuring the IREDs), the trial was repeated immediately.

After 16 trials in the first viewing condition were completed, participants were given the option to take a short break. The monocular pinhole glasses were either removed or added, and two practice trials with neutral cubes were performed under the new viewing conditions. The participant then repeated the 16 experimental trials in the same order as the first viewing condition. After the test block was completed, the calibration trials were repeated. Note that because we had fewer conditions than Maltz *et al*. [26]—two size/distance combinations rather than four—and less time was needed to transition between trials with virtual stimuli than real stimuli, we were able to double the trials per condition from 2 to 4. In summary, participants in both the VR and Real viewing conditions completed two experimental blocks (monocular and binocular viewing) with 16 trials apiece for a total of 32 experimental trials.

### (b) Results

Data for all analyses of both experiments can be accessed at https://bit.ly/3ufW7v8 (https://doi.org/10.5061/dryad.2rbnzs7rc) [38].

#### (i) Qualitative analysis of perceived size and distance

The perceived size and distance are shown for virtual objects (figure 1*b,c*) and real objects (figure 1*d*,*e*). The black squares specify the veridical sizes and distances of presented objects. The orange and red arrows represent the FSE for small/near and large/far objects, respectively (i.e. the difference in perception between objects of the same physical size and distance yet different identity).

In VR, Rubik's cubes were consistently perceived as larger than dice, regardless of their physical size and viewing condition (figure 1*b*,*c*). In other words, familiar size had a consistent, strong, influence on perceived size irrespective of the availability of binocular depth information (i.e. FSE arrows have similar lengths). This differs notably from the perception of real objects, based on the data from Maltz *et al*. [26], for which the FSE was significantly reduced when objects were viewed binocularly (figure 1*d*) rather than through a monocular pinhole (figure 1*e*; i.e. FSE arrows have shorter horizontal lengths for binocular viewing in reality). Indeed, only during binocular real-world viewing were larger objects correctly perceived as bigger in size than small objects, regardless of their identity.

Rubik's cubes were perceived as being further away than dice in all the VR conditions (figure 1*b*,*c*).

#### (ii) Statistical analysis of perceived size and distance

To confirm the qualitative observations statistically, for each of the two dependent variables (perceived size and perceived distance), we conducted a mixed four-way analysis of variance (ANOVA) with one between-subjects variable (modality [VR, real]) crossed with three within-subjects variables (viewing condition [binocular, monocular] × object identity [Rubik's cube, die] × presented size/distance [small/near, large/far]).

Because the two ANOVAs together involved 30 statistical tests (two ANOVAs, each with four main effects, six two-way interactions, four three-way interactions and one four-way interaction), to limit the likelihood of a Type II error we applied a Bonferroni correction which resulted in a *p*-value for significance testing of less than 0.0016 [39]. Interactions that reached statistical significance were further evaluated with two-tailed paired-samples *post hoc t*-tests. Because stringent correction of the ANOVAs limited the likelihood of finding interactions due to chance, we did not apply a correction for multiple comparisons on the *post hoc* tests [40]. Effect sizes were quantified using partial eta squared $\left(\eta_{p}^{2}\right)$. Statistical analyses were conducted with Jamovi (version 1.6) and JASP (version 0.16.1) statistical software. 

### (c) Perceived size

**Figure 2.**
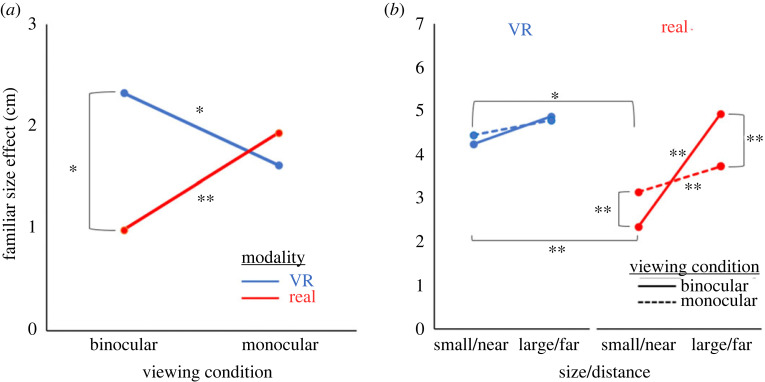
Quantitative results for size perception in experiment 1. (*a*) Interaction between object identity, modality and viewing condition on size perception. The object identity variable was collapsed into a familiar size measure (shown on the *y*-axis) by finding the difference in estimate between dice and Rubik's cubes in the same conditions. In the real-world environment, the FSE was stronger in binocular viewing than monocular viewing. By contrast, in VR, the FSE weaker in binocular viewing than monocular viewing. (*b*) Interaction between size/distance, viewing condition and modality on size perception. This interaction was driven by a greater difference in size perception between near and far objects in the binocular real condition (solid red line) than any other condition. *p < 0.05; **p < 0.001. (Online version in colour.)

The qualitative observation that the FSE depended on the combination of viewing condition and modality (figure 1) was supported statistically by a significant three-way interaction between viewing condition, modality and object identity (*F*_1,52_ = 12.3, *p* < 0.001; $\eta_{p}^{2}=0.19$). Because there was no four-way interaction, the three-way interaction is shown in figure 2*a* by plotting the FSE (the difference in perceptual estimates between Rubik's cubes and dice at the same physical size and distance, akin to the coloured arrows in figure 1) for the two viewing conditions and the two modalities. In addition, *post hoc* tests were used to further examine the interaction. Levene's test for homogeneity of variances revealed that variances for size estimates in the real and VR viewing conditions were not always equivalent. As such, we used the Welch's test (a variant of the *t*-test that is more reliable with unequal variances) for between-group comparisons when dissecting interactions involving viewing condition [41]. *t*-tests were used for *post hoc* comparisons that did not involve viewing condition.

*Post hoc* tests revealed a smaller FSE for real than VR objects during binocular viewing (Welch's *t* = 3.2, *p* = 0.002) but no difference between reality and VR during monocular pinhole viewing (Welch's *t* = 0.8, *p* = 0.42; figure 2*a*). As reported by Maltz *et al*. [26], for real objects, the FSE was stronger under monocular than binocular viewing (*t* = 3.6, *p* < 0.001; i.e. the red line in figure 2*a* has an upward slope). Surprisingly, in VR, the difference occurred in the opposite direction: the FSE was significantly stronger under binocular than monocular viewing (*t* = 2.2, *p* = 0.03; i.e. the blue line in figure 2*a* has a downward slope). One possible explanation for this reversal may be that the contribution of familiar size to perception depends not just on the information available but its reliability or congruency. That is, under binocular viewing in VR, the conflicts between vergence and accommodation may lead to a discounting of both cues and a higher weighting of familiar size.

As shown in figure 2*b*, a second significant three-way interaction was observed in size perception, involving viewing condition, modality and presented size/distance (*F*_1,52_ = 13.2, *p* < 0.001; $\eta_{p}^{2}=0.20$). This interaction was driven by a much greater difference in perceived size between small/near and large/far objects in the binocular real condition (*t* = 13.9, *p <* 0.001; solid red line in figure 2*b*) than any other condition. This finding was consistent with the qualitative finding that binocular real-world viewing emerged as the only condition where the relative presented size of the objects was perceived correctly.

### (d) Perceived distance

To evaluate the qualitative findings for distance estimates, we conducted a second mixed four-way ANOVA. The ANOVA was again Bonferroni corrected for multiple comparisons (*p* < 0.0016). The assumption of homogeneity of variances was satisfied in all between-group conditions, and thus standard *t*-tests were used to dissect significant interactions.

Perceived distance showed a main effect of object identity (familiar size) (*F*_1,52_ = 70.9, *p* < 0.001; $\eta_{p}^{2}=0.56$), which did not significantly interact with any other factors (electronic supplementary material, figure S1*a*). This shows that Rubik's cubes were perceived as being more distant than dice irrespective of presented size/distance, viewing condition or modality.

Two interactions were observed among variables other than object identity. One two-way interaction involved modality and presented size/distance (*F*_1,52_ = 15.4, *p* < 0.001; $\eta_{p}^{2}=0.23$) (electronic supplementary material, figure S1*b*). This interaction was driven by large/far objects correctly being identified as farther away than near objects in reality (*t* = 5.4, *p* < 0.001), but not in VR viewing. A second significant two-way interaction was observed between viewing condition and presented size/distance (*F*_1,52_ = 20.2, *p* < 0.001; $\eta_{p}^{2}=0.28$) (electronic supplementary material, figure S1*c*). This interaction was driven by far objects being correctly perceived as farther than near objects in binocular viewing (*t* = 4.6, *p* < 0.001), yet not in monocular viewing. These findings were consistent with qualitative results that suggested environments with salient visual cues (i.e. binocular and real-world viewing) allowed for more veridical distance perception.

### (e) Comparison of binocular virtual reality and monocular real viewing conditions

In response to a reviewer who asked whether perception differed between binocular VR and monocular real viewing conditions, we conducted between-subjects *t*-tests to contrast the FSE between these two conditions. No significant differences were found for either size (*t*_52_ = 0.76, *p* = 0.45) or distance (*t*_52_ = 0.12, *p* = 0.91).

### (f) Experiment 1 results summary

In summary, in experiment 1, we found a strong effect of familiar size on both size and distance perception in VR. Interestingly, the FSE was similar between the binocular and monocular conditions in VR, despite the availability of vergence in binocular viewing. These findings contrast with the results from the real-object study (Maltz *et al*. [26]), in which a weaker effect of familiar size was found in the binocular condition. Moreover, the FSE was comparable between the binocular VR condition and the monocular pinhole condition in reality. These results suggest that although vergence cues are available in VR (albeit with a fixed accommodation distance), they were ineffective at counteracting the powerful effect of familiar size on perception.

Experiment 1 had three limitations that we addressed in experiment 2. First, as Maltz *et al*. [26] reasoned, it is possible, albeit unlikely that the strong effect of familiar size on perception is owing to particular features of the Rubik's cube and die rather than the FSE per se. For example, Rubik's cubes and dice differ in their luminance, colour, internal geometry and associations with numbers (3 × 3 × 3 sub-cubes in a Rubik's cube; numbers of dots visible on the dice). To address this limitation, experiment 2 used a wider range of objects. Second, in experiment 1, the consumer-grade headset had limitations. Although the Oculus Rift CV-1 allows a coarse manual IPD adjustment and Unity adjusts the viewpoints from the two eyes according to IPD, this had not been individually optimized in experiment 1. With suboptimal IPD adjustments, vergence may be a less reliable cue to distance and may have led to our finding of greater reliance on familiar size in VR than in reality. Mismatches between individual IPD and and intercamera distance in VR displays can lead to distortions in reach distances [42], though it is not clear whether the relative reliance on depth cues is affected. In addition, the CV-1 had limited spatial resolution. To address this limitations, experiment 2 used a higher quality headset with optimized IPD settings and higher spatial resolution.

## 3. Experiment 2

The most likely explanation for the results of experiment 1 is that familiar size affects size and distance perception in VR regardless of the availability of oculomotor cues. Nevertheless, we conducted a second experiment to ensure that the strong reliance on FSE on perception, particularly in VR and even with full oculomotor cues available, generalized to a different category of objects and a higher quality HMD with optimized IPD settings.

**Figure 3.**
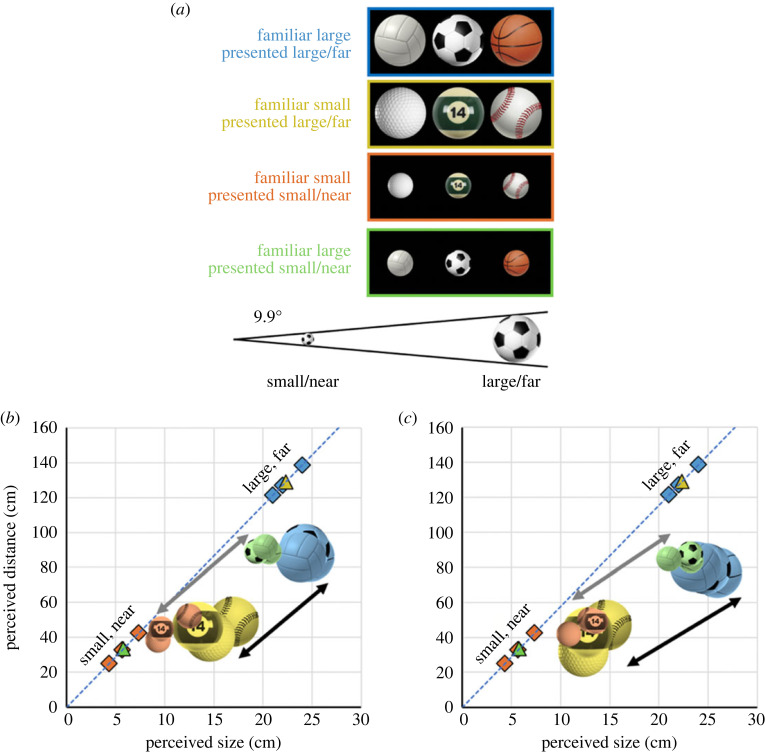
Qualitative results from experiment 2. (*a*) Three familiar large sports balls and three familiar small sports balls were presented large/far or small/near size/distance combinations, such that a retinal angle of 9.9° was maintained. (*b*,*c*) Perceived size and distance under binocular (*b*) and monocular (*c*) viewing conditions. The average perceived size and distance of each object is represented by the sports ball icons using the colour mapping indicated in (*a*). Coloured icons have been jittered slightly to reduce icon overlap. The coloured diamonds show the presented size and distance of the objects displayed at their familiar sizes and coloured triangles show the presented size and distance of the objects displayed at unfamiliar sizes. The dashed blue lines correspond to the relationship between size and distance that maintains a constant retinal angle of 9.9°. The magnitude of the FSE (difference in perception between stimuli with the same average presented size/distance) is indicated by grey arrows for the small/near objects and black arrows for the large/far objects. Note that perceived size and distance were driven largely by familiar size under both binocular and monocular viewing. (Online version in colour.)

To ensure that the results of experiment 1 were not owing to differences other than familiar size between Rubik's cubes and dice, in experiment 2, we presented six exemplars of a new class of objects—sports balls: three sports balls that are typically large (basketball, soccer ball and volleyball), and three sports balls that are typically small (baseball, pool ball and golf ball), as shown in figure 3*a*. Notably, sports balls are spheres, rather than cubes (as in experiment 1), and can have similar or different low-level properties (e.g. a golf ball and a volleyball have a comparable luminance and contrast with the background, whereas a golf ball and a basketball differ in luminance and contrast).

To assess the role that the headset properties (e.g. resolution, field of view, IPD) played in experiment 1, we used a Varjo XR-1 HMD (varjo.com) in experiment 2. The Varjo HMD automatically adjusts for individual IPD. The Varjo HMD also provides better spatial resolution through foveated rendering (60 pixels degree^−1^ versus 9.8 pixels degree^−1^ for the Oculus Rift CV-1). The enhanced resolution could improve within-object binocular disparity information and make the stimuli appear more realistic, potentially reducing the FSE under binocular viewing.

Based on the expectation that the effects of experiment 1 were owing to a strong effect of familiar size on perception in VR, and not owing to the potential confounds of the stimuli nor the properties of the HMD, we predicted that perceived size in VR would remain strongly influenced by familiar size in experiment 2.

### (a) Methods

#### (i) Participants

Twenty-four participants (age range 21–33 years) were recruited from an internal recruitment database at Western University (OurBrainsCAN Internal Pool). Otherwise, we employed the same inclusion criteria and ethics considerations as in experiment 1.

#### (ii) Apparatus and stimuli

As one of the objectives of this study was to determine if the effects seen in experiment 1 would generalize to a different class of stimuli, we presented familiar sports balls: volleyball, soccer ball, basketball (large) versus golf ball, pool ball and baseball (small). Five of the sports ball stimuli were from the Ball Sports Pack by Rivermill Studios in the Unity Asset Store, with the golf ball custom generated for a more realistic appearance. Stimuli and lighting (from point lights above the object) were optimized for rendering with Unity's high-definition render pipeline to achieve the best lighting possible in Unity.

Participants viewed the six virtual sports balls in VR using a Varjo XR-1 HMD. The Varjo HMD provides automatic adjustment for participant IPD (adjustment range 61–73 mm; supported range 59–75 mm) and uses foveated rendering to provide a resolution of 60 pixels degree^−1^ in central vision (1920 × 1080 resolution, 60-Hz refresh rate) and 16.5 pixels degree^−1^ in the periphery (1440 × 1600 screen, 87° × 87°, 90-Hz refresh rate). The XR-1 focal plane is at 1 m (John O'Neill, Varjo technical support, 2022, personal communication). Although headset weight and front-heaviness do not appear to account for perceptual distortions in mixed reality [19], one additional difference between the two studies was that the Varjo headset, which was used with a counterweight to reduce front-heaviness (1300 g), was heavier that the Oculus (470 g).

Each familiar large object was shown at its regulation size (volleyball, 21 cm, soccer ball 22 cm and basketball 24 cm) or at the average size of the familiar small objects (5.8 cm). Likewise, each familiar small object was shown at its regulation size (golf ball 4.3 cm, pool ball 5.7 cm and baseball 7.3 cm) or at the average size of the familiar large objects (22.3 cm).

As seen in figure 3*a*, the objects can therefore be divided into four groups: familiar large objects presented large/far, familiar small objects presented small/near, familiar large objects presented small/near and familiar small objects presented large/far. Objects were presented at specific distances (25.1–42.4 cm for small/near stimuli; 121–139 cm for large/far stimuli) such that the retinal image always subtended 9.9 degrees of visual angle (figure 3*b*). The balls were presented in an otherwise dark grey uniform field.

Participants manually estimated the size and distance of objects using two Valve Index VR controllers (one held in each hand), which were tracked by a Valve SteamVR 2.0 base station system. The accuracy of the controller positioning and use as an estimation tool was validated prior to testing. The program used to run this experiment and collect data was created with Unity game engine and a third-party add-in called BiomotionLab Toolkit for Unity Experiments (bmlTUX; [43]).

### (b) Procedure

On each trial, participants estimated both the size of the object and its absolute distance from them. To avoid potential order-of-estimation effects, we pseudorandomized which task was performed first. At the start of each trial, participants were told which dimension to estimate first. As in experiment 1, the stimulus was displayed as long as participants held down a button (on the controller). With a controller in each hand, participants moved their hands apart to indicate the size or distance of the stimulus. At the start of each trial, the VR controllers were held with index fingers on the trigger buttons and positioned so that their two index fingernails touched. Participants were told to use the distance between their index fingernails to make their estimates. To enter a response, participants pressed the trigger button on either of the controllers, causing the system to calculate and record the distance between the controllers, which was then used to compute the distance between the index finger locations.

After entering both estimates (size and distance), an audio cue (a single tone) sounded if the trial was successfully completed, indicating to the participant that the next trial would begin shortly. However, if an error occurred, the participant heard three tones followed by a recording of a voice describing the error and notifying the participant that they would redo the trial. Error messages were generated if a participant did not start the trial with the controllers together, rotated the controller too much during an estimate, attempted to view an object after providing only the first estimate for a trial, or took longer than 10 s to provide both estimates. The position of the VR controllers was not visible to the participant.

After participants put on the HMD and ensured it was well positioned and comfortable, they first completed two practice trials with neutral spheres. Experimental trials were blocked by two conditions (binocular viewing and monocular viewing), with order counterbalanced between participants. In binocular viewing conditions, participants viewed objects with both eyes. In monocular viewing conditions, an eyepatch covered the participant's non-dominant eye, allowing them to view objects with only their dominant eye. Unlike experiment 1, no pinhole was used because VR displays have a fixed accommodation plane and there was no need for the set-up to match the real-world conditions of Maltz *et al*. [26]. Unlike experiment 1, no chinrest was employed, although participants were discouraged from making large head movements.

For each viewing condition, participants completed 72 trials (6 objects × 2 size/distance combinations × 6 repetitions). Within blocks, trials with different objects and size/distance combinations were presented in random order.

After completing both blocks of trials, participants performed three calibration trials. While holding the VR controllers, participants positioned their hands at the ends of three wooden blocks of different length (3 cm, 45 cm and 100 cm). The system recorded the distance between the VR controllers, and the length of the appropriate wooden piece was then subtracted from this measurement to correct for individual variability in controller grasping. The six error values obtained from calibration trials were averaged, and this value was subtracted from each estimate given by the participant, thus controlling for individual variability in controller grasping.

### (c) Results

As this experiment involved six trials per condition, which is more than the previous experiments (Maltz *et al*. [26]; two trials per condition, and experiment 1: four trials per condition), for each condition and each participant, we computed the median perceptual estimates for size and distance. Using the median in lieu of the mean ensured that the condition measures taken from each participant were less vulnerable to skewing from outlier trials.

Distance and size estimates were averaged for each subject for each condition. We first qualitatively assessed the means of individuals' median perceived estimates of size and distance (by taking the mean of individual participant's medians) for the six objects at two size/distance combinations (figure 3*a*). We then validated these observations statistically using a linear mixed model analysis.

#### (i) Qualitative analysis of perceived size and distance

The results of experiment 2 are shown in figure 3 for the binocular (figure 3*b*) and monocular conditions (figure 3*c*).

Consistent with experiment 1, familiar size strongly influenced both size and distance perception. That is, the familiar large objects (blue and green icons) were always perceived as larger and farther than the familiar small objects (orange and yellow icons), regardless of presented size and distance. As such, a robust FSE, indicated by the arrows (grey arrows for small/near stimuli; black arrows for large/far stimuli) was observed under both binocular and monocular conditions (figure 3*b*,*c*).

These results agree well with the results of experiment 1. Once again, our data show that familiar size is heavily weighted in perceptual judgements of size and distance in VR, unlike in reality, where FSEs are dampened by binocular cues to bring perception closer to veridical [26].

Importantly, within familiar large and familiar small groups, object identity had a negligible effect. That is, in figure 3*b*,*c*, items of the same familiar size range and size/distance (as indicated by colour groupings) clustered tightly together, demonstrating that earlier results generalize to other stimuli.

#### (ii) Quantitative analysis of perceived size and distance

*Perceived size*. To evaluate our qualitative observations statistically, we performed two repeated-measures analyses of variance (ANOVAs)—one for perceived size and the other for perceived distance—to investigate the following independent variables and interactions: 2 [familiar size: large versus small, each collapsed across the three exemplars] × 2 [viewing condition: monocular versus binocular] × 2 [size/distance: small/near versus large/far].

The two ANOVAs together involved 14 statistical tests (two ANOVAs, each with three main effects, three two-way interactions and one three-way interaction), of which we only considered effects with *p* < 0.0035 = 0.05/14 (consistent with a Bonferroni correction) to limit the likelihood of finding any significant effect owing to chance to less than 5%.

Consistent with our qualitative observations, the ANOVA on perceived size medians revealed a large main effect of familiar size (*F*_1,23_ = 37.0, *p* < 0.001, $\eta_{p}^{2}=0.62$), supporting our prediction that size perception would depend largely on familiar size. Several trends did not survive the corrected threshold of *p* < 0.0035: a main effect of size/distance (*p* = 0.004), with large/far objects being perceived as larger than small/far objects, and an interaction of viewing condition × size/distance (*p* = 0.015), driven by a larger effect of size/distance with binocular viewing than monocular viewing. For comparisons with experiment 1, and in the spirit of a multiverse analysis to see how robust results are to analytic choices [44], we also performed an ANOVA for size perception based on means. In this analysis, the main effect of familiar size remained robust (*F*_1,23_ = 34.7, *p* < 0.001, $\eta_{p}^{2}=0.601$) but now the main effect of size/distance survived the Bonferroni-corrected threshold (*F*_1,23_ = 12.9, *p* = 0.002, $\eta_{p}^{2}=0.36$) while the interaction of size/distance and viewing condition did not (*p* = 0.07). In summary, regardless of the use of means or medians, a large FSE for size perception was found and in no case did familiar size (i.e. object identity) interact with size/distance or viewing condition.

To demonstrate that perceived size was consistent with familiar size across all six exemplars, we examined the correlation between familiar size and perceived size (based on group averages for each). This correlation (electronic supplementary material, figure S2*a*) accounted for most of the variance in the data (monocular viewing: *r* = 0.98, *p* < 0.001; binocular viewing: *r* = 0.90, *p* < 0.001) with no objects appearing as outliers along this function. By contrast, the correlation between presented size and perceived size (electronic supplementary material, figure S2*b*) accounted for relatively little variance in the data (monocular viewing: *r* = 0.14, *p* = 0.65; binocular viewing: *r* = 0.42, *p* = 0.18).

*Perceived distance*. Consistent with our qualitative observations, the 2 × 2 × 2 ANOVA on perceived distance medians found only a main effect of familiar size (*F*_2,23_ = 54.0, *p* < 0.001, $\eta_{p}^{2}=0.7$) with no other significant main effects or interactions (all *p* > 0.1). An analysis of means was fully consistent.

To demonstrate that perceived size was consistent with familiar size across all six exemplars, we examined the correlation between the distance that would be consistent with familiar size (= familiar size/tan (9.9°)) and perceived size (based on group averages for each). This correlation (electronic supplementary material, figure S2*c*) accounted for most of the variance in the data (monocular viewing: *r* = 0.97, *p* < 0.001; binocular viewing: *r* = 0.99, *p* < 0.001), with no objects appearing as outliers along this function. By contrast, the correlation between presented distance and perceived distance (electronic supplementary material, figure S2*d*) accounted for relatively little variance in the data (monocular viewing: *r* = −0.16, *p* = 0.62; binocular viewing: *r* = −0.03, *p* = 0.93).

## 4. Discussion

The primary objective of this study was to investigate how size and distance perception in VR were affected by familiar size, which could either be consistent or inconsistent with presented size/distance. In experiment 1, we compared size and distance perception in VR with results from an earlier experiment that used tangible versions of the same stimuli in a physical set-up [26]. We found a strong FSE in VR during both monocular and binocular viewing; that is, size and distance perception were strongly biased towards familiar size even when a binocular cue (vergence) might have provided information about the presented distance, which in turn might have enabled inference of the presented size. Importantly, the FSE for size perception was stronger in binocular VR viewing than binocular real-world viewing. Indeed, binocular real-world viewing emerged as the only condition where the perceived stimulus size was more strongly influenced by the presented size/distance than by familiar size. Familiar size had a comparable effect on distance perception regardless of whether stimuli were presented in VR or physically and whether they were presented binocularly or through a monocular pinhole. Notably, the FSE for both familiar sizes did not differ statistically between binocular viewing in VR and monocular pinhole viewing in the real world. In experiment 2, we showed that the strong contribution of familiar size found in experiment 1 generalized to another class of objects (six familiar sports balls) when viewed in an HMD with a larger field of view, improved resolution, and IPDs that were optimized for individual participants. Taken together, these results indicate that the relative importance of familiar size versus presented size/distance for size and distance perception differs between VR and physical environments, regardless of object identity or HMD quality.

### (a) The perception of virtual reality and physical stimuli have different dependencies on familiar size and presented size/distance

These results are consistent with previous results showing that the relative weighting of sensory information is malleable [45,46] and affected by cue reliability [47–51]. Experiments using highly controlled conditions to isolate specific cues to depth have shown that depth judgements are well described by Bayesian approaches to cue integration for binocular disparity and texture [52,53], and motion [54,55]. Perhaps because of the influence of cue reliability, there is also good evidence that the combination of depth information from different sources is affected by cue conflicts and, for virtual stimuli, display-based conflicts [56–58]. The few studies that have used physical stimuli have also found evidence for differential cue weighting [39–41], and under some conditions, vetoing of less reliable cues [56,59]. There is debate concerning the applicability and assumptions of the Bayesian approach to cue combination, for instance the assumption of cue independence [60] which is beyond the scope of this paper. However, the up- or down-weighting of information based on context or reliability is widely accepted.

The reliability of familiar size information would not be expected to change in VR (relative to the real world) as long as there is sufficient display resolution; however, our results suggest that poorer reliability of oculomotor cues in VR evokes a relatively greater reliance on familiar size. By contrast, in the physical test environment, the availability of both vergence and accommodation in the binocular viewing condition increased the contribution of presented size/distance to size perception and decreased the contribution of familiar size. In VR, the ability to change vergence under binocular viewing did not affect the contribution of the displayed size/distance information to size perception and, surprisingly, increased the contribution of familiar size. This increased dependence on familiar size in the VR condition might also have been enhanced because accommodation was fixed at the focal plane of the display so under some conditions (e.g. when the observer converged to fixate on a near object), there was a significant conflict between vergence and accommodation.

Because of their geometric relationship, the retinal angle and distance of an object can be used to compute its size. As such, we might expect that size and distance would be similarly impacted by the distance cues of familiar size and vergence [61]. Surprisingly however, the perception of size and distance are weighted differently by these factors. That is, the effect of familiar size on distance estimates changed little across conditions; by contrast, the effect of familiar size on size estimates depended both on modality and viewing condition.

### (b) Why does perception differ between virtual reality and the physical world?

It is clear from our results that familiar size and presented size have different impacts on perception in VR and reality; why does this occur? The obvious difference between the two viewing environments is that in the physical environment, binocular viewing provides coordinated information from both vergence and accommodation and these two cues are consistent. By contrast, with binocular viewing in VR, accommodation does not provide veridical information (because the images are always presented at the focal distance of the screens); thus, for any virtual distances outside the focal plane, accommodation is in conflict with vergence and accommodative blur is unavailable. There is a long history of debate concerning the relative contributions of vergence and accommodation as cues to distance. Helmholtz believed that vergence was used to gauge egocentric distance [62]; since then, numerous studies have shown that vergence can help support distance estimation, particularly under natural viewing conditions where it is supported by other information such as horizontal and vertical disparities [28,29,63] and accommodation [64–66]. However, under reduced conditions where vergence changes occur in isolation, it is a relatively weak cue to distance [67]; in the extreme, when all other sources of information (including binocular disparity) are removed, Linton [30] has shown that there is negligible impact of vergence on perceived distance [30]. In such cases of impoverished viewing conditions, observers have been shown to assume a default distance. This ‘specific distance tendency’ varies across observers but is approximately 2 m and in early experiments has been shown to affect size judgements [68]. Because it is difficult to isolate it from vergence, the effect of accommodation changes on distance perception is poorly documented. For instance, Mon-Williams & Tresilian [69] found that accommodation in isolation provided very poor distance information but that it did seem to bias some observers' responses [69]. In that study, they allow that the bias may have been driven by an accommodative vergence response. Fisher & Ciuffreda [70] were able to document changes in apparent distance owing to changes in accommodation but only in near space (around 30 cm) and only for high-contrast patterns [70,71]. This result is consistent with reviews of size and depth constancy in showing that veridical scaling of this information is limited to distances less than 1 m where we are most sensitive to vergence and accommodative changes either in isolation or in combination [53,54]. Studies have examined the impact of blur on depth perception (as a proxy for accommodation) and shown that it can be a strong source of relative depth information [72,73], but stimulus blur is not useful as a cue to absolute distance.

A limited number of studies have examined the effects of conflicts between vergence and accommodation on depth or distance perception. There is some consensus that VAC disrupts depth discrimination [6,7,74] and distance perception [75,76] within near space for individuals with normal accommodative responses (and without presbyopia). There is a much larger body of work that shows that VAC leads to discomfort and fatigue [77–79]. These considerations have led to intense interest in the development of multi-focal 3D displays and VR headsets [80,81]. A full treatment of this area is beyond the scope of this paper, but for a review see Zhan *et al*. [82]. While these technical advances are promising, further research will be needed to determine if elimination of VAC will restore veridical size/distance perception.

Although the VAC is the most obvious limitation of VR, other distortions should also be considered. As reviewed elsewhere [83], current VR displays do not perfectly match the visual information provided in natural environments for many reasons. Our results are not easily explained by a limited field of view [16,19] because the objects were in central vision, surrounded by a black void nor display latencies [84] (because head movements and motion parallax were limited). However, other limitations of VR could have plausibly affected our results. Most notably, VR has imperfect geometries owing to optical distortions [83] (including ‘pupil swim’ owing to lens distortions with eye movements). Such distortions may have reduced the reliability and weighting of low-level cues to distance in favour of the high-level cue of familiar size. Although experiment 2 suggests that the spatial resolution of the displays had little effect, as the objects were clearly defined, resolution is not only important for the crisp appearance of the stimulus but also for rendering fine binocular disparities. Other distortions may arise from the nature of stimulus development software like Unity, which was developed for fast latencies in gaming rather than verisimilitude in research. In addition, there may have been other high-level factors that changed perception, including increased motion sickness in VR displays, fatigue from the VAC [85], and the reduced realism and sense of presence from virtual objects compared to real ones [86,87]. Some of these factors may be reduced or eliminated as VR technology improves, particularly if systems become more optically accurate through better calibration or if systems using optically veridical ray-traced images can be successfully developed.

### (c) Strategies for improving accuracy of perception in virtual reality

Although the enhanced reliance on familiar size in VR could be seen as problematic for VR applications that require veridical perception (e.g. image-guided surgery), familiar size could be used as a strategic tool for improving size and distance perception in VR. Indeed, participants were substantially better at determining the sizes and distances of blank spheres when they were displayed beside a familiar object (a milk carton) [88]. Moreover, the perceived size of unfamiliar objects is even more affected by the size of one's own hand rendered as an avatar seen from the first-person perspective than by a familiar object [33]

Perception in VR may also be improved by providing participants with opportunities to improve through feedback about accuracy (e.g. a tone with pitch indicating errors or their magnitude) or sensorimotor consequences (e.g. seeing whether a reach-to-grasp action achieves an appropriate distance and grip size for the object) [89,90]. Notably, our participants never received any feedback about their performance. Research has shown that performance in VR can be improved by feedback [91–94].

### (d) Limitations

Although our results suggest that VR is not a perfect proxy for the physical world, we should also acknowledge that our display was impoverished compared to true reality. Most notably, our environment consisted only of single objects lacking context, such as a ground plane and other objects, a full-field environment that could provide vertical disparities, and visuomotor feedback that could help the visual system to calibrate the reliability of information sources. Indeed, past studies of VR have found that rich environments with many objects lead to more accurate size perception [20,21]. Nevertheless, our paradigm and results provide an example of how the contributions of multiple cues can be evaluated across different levels of realness and stress the importance of benchmarking processing in VR not only against simpler stimuli (such as flat images) but also against true reality [1]. One interesting domain for future research is augmented reality systems, in which virtual images are presented (with vergence but a fixed accommodation plane as in VR) superimposed on real scenes (with full-depth cues). Based on our results here, we would predict that perception of virtual images would be more strongly affected by the FSE compared to real objects in such mixed reality displays.

Another limitation of our study is that our dependent measure—delayed manual estimation—relies on participants' perception and memory of size and distance. Our results may have differed had we had participants judge size and distance while the stimuli were still visible. Our results may have also differed had we had participants perform action tasks such as grasping. One theoretical view is that tasks involving perception and memory rely more on the ventral visual stream while tasks involving online actions (such as grasping) rely more on the dorsal visual stream [95,96]. Intriguing evidence from a neuropsychological patient suggests that action tasks tap into information about distance and size that are inaccessible to perception tasks [97], a result consistent with other evidence that areas of the dorsal ‘vision for action’ stream process information about distance from vergence [98]. Studies that have employed action tasks such as reaching or grasping with real objects have found that with neutral objects, binocular cues dominate performance [99], while those using familiar objects have found influences of familiar size [100]. Studies with reaching towards virtual objects have found contributions of both binocular vision and familiar size affect distance perception [101,102]. Moreover, the contribution of stereopsis was stronger when participants reached with their eyes open versus eyes closed after object presentation, whereas the contribution of familiar size was stronger during reaching with eyes closed versus open [101,102]. Ideally, future studies of the accuracy of size and distance processing for action tasks in VR will be more tailored to the tasks typically performed in VR (e.g. gaming, image-guided surgery) and could inform the debate as to how well VR invokes realistic action processing [98,103].

## 5. Conclusion

Our results contribute to a theoretical understanding of vision by extending past research on differences in cue weighting between VR and physical reality to incorporate familiar size. Specifically, our findings demonstrate that familiar size is an especially potent cue in VR, presumably because of the absence of accommodation, the conflict between vergence and accommodation, and/or other distortions in VR.

Our results also inform the practical implementation of VR for research and applications (such as image-guided surgery and surgical training). The findings suggest that caution is warranted in assuming that results from VR will necessarily generalize to the real world. Moreover, they suggest that further research is needed to determine whether these differences can be mitigated by better technologies or by developing better strategies for virtual environments.

Growing evidence suggests substantial differences in behaviour and brain processing for real (tangible) objects compared to two-dimensional images [1]. The hope has been that VR can provide the ‘best of both worlds’: the realism of the physical world along with the experimental control of the laboratory world. Researchers take different theoretical perspectives about the degree to which VR can serve as a proxy for reality [103] or not [5]. This debate has often centred on the use of VR to study vision for action, where 3D vision and realness may be particularly important [104–106]. However, even our purely perceptual task reveals substantial differences.

## Data Availability

The data are available from the Dryad Digital Repository: https://doi.org/10.5061/dryad.2rbnzs7rc [38]. The data are provided in the electronic supplementary material [107].

## References

[1] Snow JC, Culham JC. 2021 The treachery of images: how realism influences brain and behavior. Trends Cogn. Sci. **25**, 506-519. (10.1016/j.tics.2021.02.008) 33775583 PMC10149139

[2] Loomis J, Blascovich J, Beall A. 1999 Immersive virtual environment technology as a basic research tool in psychology. Presence Interaction Shared Virtual Environ. **31**, 557-564. (10.3758/bf03200735) 10633974

[3] Zaal FTJM, Bootsma RJ. 2011 Virtual reality as a tool for the study of perception-action: the case of running to catch fly balls. Presence: Teleoperators and Virtual Environments **20**, 93-103. (10.1162/pres_a_00037)

[4] Glas HH, Kraeima J, van Ooijen PMA, Spijkervet FKL, Yu L, Witjes MJH. 2021 Augmented reality visualization for image-guided surgery: a validation study using a three-dimensional printed phantom. J. Oral Maxillofac. Surg. **79**, 1943.e1-1943.e10. (10.1016/j.joms.2021.04.001) 34033801

[5] Harris DJ, Buckingham G, Wilson MR, Vine SJ. 2019 Virtually the same? How impaired sensory information in virtual reality may disrupt vision for action. Exp. Brain Res. **237**, 2761-2766. (10.1007/s00221-019-05642-8) 31485708 PMC6794235

[6] Hoffman DM, Banks MS. 2010 Vergence–accommodation conflicts hinder visual performance and cause visual fatigue. J. Vis. **8**, 33. (10.1167/8.3.33.Vergence) PMC2879326 18484839

[7] Kim J, Kane D, Banks MS. 2014 The rate of change of vergence-accommodation conflict affects visual discomfort. Vision Res. **105**, 159-165. (10.1016/j.visres.2014.10.021.The) 25448713 PMC4294985

[8] Witmer BG, Sadowski WJ. 1998 Nonvisually guided locomotion to a previously viewed target in real and virtual environments. Hum. Factors **40**, 478-488. (10.1518/001872098779591340)

[9] Armbrüster C, Wolter M, Kuhlen T, Spijkers W, Fimm B. 2008 Depth perception in virtual reality: DISTANCE estimations in peri- and extrapersonal space. Cyberpsychol. Behav. **11**, 9-15. (10.1089/cpb.2007.9935) 18275307

[10] Stefanucci JK, Creem-Regehr SH, Thompson WB, Lessard DA, Geuss MN. 2015 Evaluating the accuracy of size perception on screen-based displays: displayed objects appear smaller than real objects. J. Exp. Psychol. **21**, 215-223. (10.1037/xap0000051) 26121374

[11] Kelly JW, Cherep LA, Klesel B, Siegel ZD, George S. 2018 Comparison of two methods for improving distance perception in virtual reality. ACM Trans. Appl. Percept. **15**, 1-12. (10.1145/3165285)

[12] Messing R, Durgin FH. 2005 Distance perception and the visual horizon in head-mounted displays. ACM Trans. Appl. Percept. **2**, 234-250. (10.1145/1077399.1077403)

[13] Wann JP, Rushton S, Mon-Williams M. 1995 Natural problems for stereoscopic depth perceptions in virtual environments. Vision Res. **35**, 2731-2736. (10.1016/0042-6989(95)00018-U) 7483313

[14] Witmer BG, Kline PB. 1998 Judging perceived and traversed distance in virtual environments. Presence: Teleoperators and Virtual Environments **7**, 144-167. (10.1162/105474698565640)

[15] Knapp JM, Loomis JM. 2004 Limited field of view of head-mounted displays is not the cause of distance underestimation in virtual environments. Presence: Teleoperators and Virtual Environments **13**, 572-577. (10.1162/1054746042545238)

[16] Creem-Regehr SH, Willemsen P, Gooch AA, Thompson WB. 2005 The influence of restricted viewing conditions on egocentric distance perception: implications for real and virtual indoor environments. Perception **34**, 1-2. (10.1068/p5144) 15832569

[17] Jones JA, Swan JA, Bolas M. 2013 Peripheral stimulation and its effect on perceived spatial scale in virtual environments. IEEE Trans. Vis. Comput. Graph. **19**, 701-710. (10.1109/TVCG.2013.37) 23428455

[18] Willemsen P, Colton MB, Creem-Regehr SH, Thompson WB. 2009 The effects of head-mounted display mechanical properties and field of view on distance judgments in virtual environments. ACM Trans. Appl. Percept. **6**, 1-14. (10.1145/1498700.1498702)

[19] Gagnon HC, Zhao Y, Richardson M, Pointon GD, Stefanucci JK, Creem-Regehr SH, Bodenheimer B. 2021 Gap affordance judgments in mixed reality: testing the role of display weight and field of view. Front. Virtual Reality **2**, 1-15. (10.3389/frvir.2021.654656)

[20] Kenyon R, Sandin D, Smith RC, Pawlicki R, Defanti T. 2007 Size-constancy in the CAVE. Presence: Teleoperators and Virtual Environments **16**, 172-187. (10.1162/pres.16.2.172)

[21] Luo X, Kenyon R, Kamper D, Sandin D, DeFanti T. 2007 The effects of scene complexity, stereovision, and motion parallax on size constancy in a virtual environment. In Proc. IEEE Virtual Reality, *10–14 March 2007, Charlotte, NC, USA*, pp. 59–66. New York, NY: IEEE.

[22] Berkeley G. 1709 An essay towards a new theory of vision (based on 1st edn, edited by DR Wilkins, 2002). Dublin, Ireland: Jeremy Pepyat.

[23] Bolles RC, Bailey DE. 1956 Importance of object recognition in size constancy. J. Exp. Psychol. **51**, 222-225. (10.1037/h0048080) 13306868

[24] Fitzpatrick V, Pasnak R, Tyer ZE. 1982 The effect of familiar size at familiar distances. Perception **11**, 85-91. (10.1068/p110085) 7133938

[25] Schiffman HR. 1967 Size-estimation of familiar objects under informative and reduced conditions of viewing. Am. J. Psychol. **80**, 229-235. (10.2307/1420981) 6055054

[26] Maltz MV, Stubbs KM, Rzepka AM, Martin JR, Culham JC. 2021 Familiar size affects the perceived size and distance of real objects even with binocular vision. J. Vis. **21**, 1-18. (10.1167/jov.21.10.21) PMC8479574 34581767

[27] Bradshaw MF, Glennerster A, Rogers BJ. 1996 The effect of display size on disparity scaling from differential perspective and vergence cues. Vision Res. **36**, 1255-1264. (10.1016/0042-6989(95)00190-5) 8711905

[28] Glennerster A, Rogers BJ, Bradshaw MF. 1998 Cues to viewing distance for stereoscopic depth constancy. Perception **27**, 1357-1365. (10.1068/p271357) 10505180

[29] Mon-Williams M, Tresilian JR, Roberts A. 2000 Vergence provides veridical depth perception from horizontal retinal image disparities. Exp. Brain Res. **133**, 407-413. (10.1007/s002210000410) 10958531

[30] Linton P. 2020 Does vision extract absolute distance from vergence? Attention Percept. Psychophys. **82**, 3176-3195. (10.3758/s13414-020-02006-1) PMC7381460 32406005

[31] Distler HK, Gegenfurtner KR, van Veen HAHC, Hawken MJ. 2000 Velocity constancy in a virtual reality environment. Perception **29**, 1423-1435. (10.1068/p3115) 11257966

[32] Nguyen TD, Ziemer CJ, Grechkin T, Chihak B, Plumert JM, Cremer JF, Kearney JK. 2011 Effects of scale change on distance perception in virtual environments. ACM Trans. Appl. Percept. **8**, 1-18. (10.1145/2043603.2043608)

[33] Linkenauger SA, Leyrer M, Bülthoff HH, Mohler BJ. 2013 Welcome to wonderland: the influence of the size and shape of a virtual hand on the perceived size and shape of virtual objects. PLoS ONE **8**, e68594. (10.1371/journal.pone.0068594) 23874681 PMC3708948

[34] Goodale MA, Jakobsonp LS, Keillors JM. 1994 Differences in the visual control of pantomimed and natural grasping movements. Neuropsychologia **32**, 1159-1178. (10.1016/0028-3932(94)90100-7) 7845558

[35] Hartle B, Wilcox LM. 2016 Depth magnitude from stereopsis: assessment techniques and the role of experience. Vision Res. **125**, 64-75. (10.1016/j.visres.2016.05.006) 27369096

[36] Kopiske KK, Domini F. 2018 On the response function and range dependence of manual estimation. Exp. Brain Res. **236**, 1309-1320. 29502246 10.1007/s00221-018-5223-5

[37] Oldfield R. 1971 The assessment and analysis of handedness: The Edinburgh inventory. Neuropsychologia **9**, 97-113. (10.1016/0028-3932(71)90067-4) 5146491

[38] Rzepka AM, Hussey KJ, Maltz MV, Babin K, Wilcox LM, Culham JC. 2022 Data from: Familiar size effects perception differently in virtual reality and the real world. Dryad Digital Repository. (10.5061/dryad.2rbnzs7rc) PMC9745877 36511414

[39] Cramer AOJ, van Ravenzwaaij D, Matzke D, Steingroever H, Wetzels R, Grasman RPPP, Waldorp LJ, Wagenmakers EJ. 2016 Hidden multiplicity in exploratory multiway ANOVA: PREVALENCE and remedies. Psychonomic Bullet. Rev. **23**, 640-647. (10.3758/s13423-015-0913-5) PMC4828473 26374437

[40] Rosenthal R, Rosnow R. 1991 Essentials of behavioral research: methods and data analysis, 2nd ed. New York, NY: McGraw-Hill.

[41] Welch BL. 1947 The generalisation of student's problems when several different population variances are involved. Biometrika **34**, 28-35. (10.1093/biomet/34.1-2.28) 20287819

[42] Renner RS, Steindecker E, Müller M, Velichkovsky BM, Stelzer R, Pannasch S, Helmert JR. 2015 The influence of the stereo base on blind and sighted reaches in a virtual environment. ACM Trans. Appl. Percept. **12**, 1-18. (10.1145/2724716)

[43] Bebko AO, Troje N. 2020 bmlTUX: design and control of experiments in virtual reality and beyond. Iperception **11**, 2041669520938400. 32733664 10.1177/2041669520938400 PMC7370570

[44] Steegen S, Tuerlinckx F, Gelman A, Vanpaemel W. 2016 Increasing transparency through a multiverse analysis. Perspect. Psychol. Sci. **11**, 702-712. (10.1177/1745691616658637) 27694465

[45] Scarfe P, Glennerster A. 2021 Combining cues to judge distance and direction in an immersive virtual reality environment. J. Vis. **21**, 1-25. (10.1167/JOV.21.4.10) PMC8083085 33900366

[46] Svarverud E, Gilson SJ, Glennerster A. 2010 Cue combination for 3D location judgements. J. Vis. **10**, 1-13. (10.1167/10.1.5) PMC2836116 20143898

[47] Landy M, Maloney L, Johnston E, Young M. 1995 Measurement and modeling of depth cue combination: in defense of weak fusion. Vision Res. **35**, 389-412. (10.1016/0042-6989(94)00176-m) 7892735

[48] Knill DC, Pouget A. 2004 The Bayesian brain: the role of uncertainty in neural coding and computation. Trends Neurosci. **27**, 712-719. (10.1016/j.tins.2004.10.007) 15541511

[49] Bankieris KR, Bejjanki VR, Aslin RN. 2017 Sensory cue-combination in the context of newly learned categories. Sci. Rep. **7**, 1-10. (10.1038/s41598-017-11341-7) 28883455 PMC5589839

[50] Maloney LT, Landy MS. 1989 A statistical framework for robust fusion of depth information. In Proc. SPIE 1199, Visual Communications and Image Processing IV, Philadelphia, PA, 1989. Paris, France: Society of Photo-Optical Instrumentation Engineers (SPIE).

[51] Glennerster A, Tcheang L, Gilson SJ, Fitzgibbon AW, Parker AJ. 2006 Humans ignore motion and stereo cues in favor of a fictional stable world. Curr. Biol. **16**, 428-432. (10.1016/j.cub.2006.01.019) 16488879 PMC2833396

[52] Knill DC, Saunders JA. 2003 Do humans optimally integrate stereo and texture information for judgments of surface slant? Vision Res. **43**, 2539-2558. (10.1016/S0042-6989(03)00458-9) 13129541

[53] Hillis JM, Watt SJ, Landy MS, Banks MS. 2004 Slant from texture and disparity cues: optimal cue combination. J. Vis. **4**, 967-992. (10.1167/4.12.1) 15669906

[54] Landy M, Brenner E. 2001 Motion-disparity interaction and the scaling of stereoscopic disparity. In Vision and attention (eds M Jenkin, L Harris), pp. 129-150. New York, NY: Springer.

[55] Johnston EB, Cumming BG, Landy MS. 1994 Integration of stereopsis and motion shape cues. Vision Res. **34**, 2259-2275. (10.1016/0042-6989(94)90106-6) 7941420

[56] Norman JF, Todd JT. 1995 The perception of 3-D structure from contradictory optical patterns. Percept. Psychophys. **57**, 826-834. (10.3758/BF03206798) 7651807

[57] Todd JT, Norman JF. 2003 The visual perception of 3-D shape from multiple cues: are observers capable of perceiving metric structure? Percept. Psychophys. **65**, 31-47. (10.3758/BF03194781) 12699307

[58] Scarfe P, Hibbard PB. 2011 Statistically optimal integration of biased sensory estimates. J. Vis. **11**, 1-17. (10.1167/11.7.1) 21670095

[59] Girshick AR, Banks MS. 2009 Probabilistic combination of slant information: weighted averaging and robustness as optimal percepts. J. Vis. **9**, 1-20. (10.1167/9.9.8) 19761341 PMC2940417

[60] Domini F, Caudek C, Tassinari H. 2006 Stereo and motion information are not independently processed by the visual system. Vision Res. **46**, 1707-1723. (10.1016/j.visres.2005.11.018) 16412492

[61] Gibson JJ. 1972 A theory of direct visual perception. In Vision and mind: selected readings in the philosophy of perception (eds A Noe, E Thompson), pp. 77-90. New York, NY: MIT Press.

[62] Helmholtz Hv. 1962 Treatise on physiological optics (translated from handbuch der physiologischen optik, 1910 by southall JPC), vol. 3. New York, NY: Dover Publications.

[63] Rogers B, Bradshaw M. 1995 Disparity scaling and the perception of frontoparallel surfaces. Perception **24**, 155-179. (10.1068/p240155) 7617423

[64] Gogel WC. 1977 An indirect measure of perceived distance from oculomotor cues. Percept. Psychophys. **21**, 3-11. (10.3758/BF03199459)

[65] Wallach H, Zuckerman C. 1963 The constancy of stereoscopic depth. Am. J. Psychol. **76**, 404-412. (10.2307/1419781) 13998575

[66] Foley J. 1980 Binocular distance perception. Psychol. Rev. **87**, 411-434. (10.1037/0033-295X.87.5.411) 7413886

[67] Richards W, Miller JF. 1969 Convergence as a cue to depth. Percept. Psychophys. **5**, 317-320. (10.3758/BF03209573)

[68] Gogel WC. 1969 The sensing of retinal size. Vision Res. **9**, 1079-1094. (10.1016/0042-6989(69)90049-2) 5350376

[69] Mon-Williams M, Tresilian JR. 2000 Ordinal depth information from accommodation? Ergonomics **43**, 391-404. (10.1080/001401300184486) 10755661

[70] Fisher S, Ciuffreda K. 1988 Accommodation and apparent distance. Perception **17**, 609-621. (10.1068/p170609) 3249669

[71] Mon-Williams M, Tresilian JR. 1999 Some recent studies on the extraretinal contribution to distance perception. Perception **28**, 167-181. (10.1068/p2737) 10615458

[72] Vishwanath D. 2012 The utility of defocus blur in binocular depth perception. Iperception **3**, 541-546. (10.1068/i0544ic) 23145307 PMC3485857

[73] Held RT, Cooper EA, Banks MS. 2012 Blur and disparity are complementary cues to depth. Curr. Biol. **22**, 426-431. (10.1016/j.visres.2014.10.036) 22326024 PMC3298574

[74] Vienne C, Masfrand S, Bourdin C, Vercher JL. 2020 Depth perception in virtual reality systems: effect of screen distance, environment richness and display factors. IEEE Access **8**, 29 099-29 110. (10.1109/ACCESS.2020.2972122)

[75] Kunnapas T. 1968 Distance perception as a function of available visual cues. J. Exp. Psychol. **7**, 523-529. (10.1037/h0026050) 5672260

[76] Vienne C, Blondé L, Mamassian P. 2015 Depth-of-focus affects 3D perception in stereoscopic displays. Perception **44**, 613-627. (10.1177/0301006615594261) 26489206

[77] Lambooij M, Fortuin M, Heynderickx I, IJsselsteijn W. 2009 Visual discomfort and visual fatigue of stereoscopic displays: a review. J. Imaging Sci. Technol. **53**, 30201-1-30201-14. (10.2352/j.imagingsci.technol.2009.53.3.030201)

[78] Shibata T, Kim J, Hoffman DM, Banks MS. 2011 The zone of comfort: predicting visual discomfort with stereo displays. J. Vis. **11**, 1-29. (10.1167/11.8.1) PMC3369815 21778252

[79] Kooi FL, Toet A. 2004 Visual comfort of binocular and 3D displays. Displays **25**, 99-108.

[80] Akeley K, Watt SJ, Girschick AR, Banks M. 2004 A stereo display prototype with multiple focal distances. ACM Trans. Graphic **23**, 804-813.

[81] Rolland JP, Krueger MW, Goon A. 2000 Multifocal planes head-mounted displays. Appl. Opt. **39**, 3209-3215. 18349886 10.1364/ao.39.003209

[82] Zhan T, Xiong J, Zou J, Wu S. 2020 Multifocal displays: review and prospect. PhotoniX **1**, 1-31. (10.1186/s43074-020-00010-0)

[83] Scarfe P, Glennerster A. 2019 The science behind virtual reality displays. Annu. Rev. Vision Sci. **5**, 529-547. (10.1146/annurev-vision-091718-014942) 31283449

[84] Glennerster A, Gilson S. 2017 Measuring end-to-end latency of a virtual reality system objectively and psychophysically. Vision Sci. Soc. Annu. Meeting Abstract **17**, 355. (10.1167/17.10.355)

[85] Shibata T, Kim J, Hoffman DM, Banks MS. 2011 Visual discomfort with stereo displays: effects of viewing distance and direction of vergence-accommodation conflict. Proc. SPIE **7863**, 78630P1-78630P9. (10.1117/12.872347) PMC3150963 21826254

[86] Sanchez-Vives Mv, Slater M. 2005 From presence to consciousness through virtual reality. Nat. Rev. Neurosci. **6**, 332-339. (10.1038/nrn1651) 15803164

[87] Renshaw TJ, Sonnenfeld NA, Meyers MD. 2016 Fundamentals for a Turing test of virtual reality [abstract]. Proc. Human Factors Ergonomics Soc. Annu. Meeting **60**, 2113-2117. (10.1177/1541931213601478)

[88] Lammi J, Moilanen P, Sierilä A. 2019 Size and distance estimation in virtual reality. Bachelor's thesis, University of Oulu, Oulu, Finland. See http://jultika.oulu.fi/files/nbnfioulu-201906132541.pdf.

[89] Whitwell RL, Katz NJ, Goodale MA, Enns JT. 2020 The role of haptic expectations in reaching to grasp: from pantomime to natural grasps and back again. Front. Psychol. **11**, 1-16. (10.3389/fpsyg.2020.588428) 33391110 PMC7773727

[90] Coats R, Bingham GP, Mon-Williams M. 2008 Calibrating grasp size and reach distance: interactions reveal integral organization of reaching-to-grasp movements. Exp. Brain Res. **189**, 211-220. (10.1007/s00221-008-1418-5) 18493753

[91] Fulvio JM, Rokers B. 2017 Use of cues in virtual reality depends on visual feedback. Sci. Rep. **7**, 1-13. (10.1038/s41598-017-16161-3) 29167491 PMC5700175

[92] Mohler BJ, Creem-Regehr SH, Thompson WB. 2006 The influence of feedback on egocentric distance judgments in real and virtual environments. In Proc. APGV 2006: Symp. on Applied Perception in Graphics and Visualization, *28–29 July 2006, Boston, MA,* vol. 1. New York, NY: Association for Computing Machinery.

[93] Gagnon HC, Rohovit T, Finney H, Yu Z, Franchak JM. 2021 The effect of feedback on estimates of reaching ability in virtual reality. In IEEE Conf. on Virtual Reality and 3D User Interfaces, virtual, 27 March–2 April 2021. New York, NY: IEEE.

[94] Gagnon HC, Na D, Heiner K, Stefanucci J, Creem-Regehr S. 2020 The role of viewing distance and feedback on affordance judgments in augmented reality. In IEEE Conf. on Virtual Reality and 3D User Interfaces. New York, NY: IEEE.

[95] Goodale MA, Milner DA. 1992 Separate visual pathways for perception and action. Trends Neurosci. **15**, 20-25. (10.1016/0166-2236(92)90344-8) 1374953

[96] Goodale MA, Westwood DA, Milner AD. 2004 Two distinct modes of control for object-directed action. Prog. Brain Res. **144**, 131-144. (10.1016/s0079-6123(03)14409-3) 14650845

[97] Whitwell RL, Sperandio I, Buckingham G, Chouinard PA, Goodale MA. 2020 Grip constancy but not perceptual size constancy survives lesions of early visual cortex. Curr. Biol. **30**, 3700-3701. (10.1016/j.cub.2020.08.025) 32961148

[98] Quinlan DJ, Culham JC. 2007 fMRI reveals a preference for near viewing in the human parieto-occipital cortex. Neuroimage **36**, 167-187. (10.1016/j.neuroimage.2007.02.029) 17398117

[99] Marotta JJ, Goodale MA. 2001 The role of familiar size in the control of grasping. J. Cogn. Neurosci. **13**, 8-17. (10.1162/089892901564135) 11224905

[100] McIntosh RD, Lashley G. 2008 Matching boxes: familiar size influences action programming. Neuropsychologia **46**, 2441-2444. (10.1016/j.neuropsychologia.2008.03.003) 18407302

[101] Schubert RS, Müller M, Pannasch S, Helmert JR. 2016 Depth information from binocular disparity and familiar size is combined when reaching towards virtual objects. In Proc. of the ACM Symp. on Virtual Reality Software and Technology, VRST, 2–4 November 2016, Munich, Germany, pp. 233-236. New York, NY: Association for Computing Machinery.

[102] Schubert RS, Jung ML, Helmert JR, Velichkovsky BM, Pannasch S. 2019 Size matters: how reaching and vergence movements are influenced by the familiar size of stereoscopically presented objects. PLoS ONE **14**, 1-29. (10.1371/journal.pone.0225311) PMC6867642 31747431

[103] Troje NF. 2019 Reality check. Perception **48**, 1033-1038. (10.1177/0301006619879062) 31570079

[104] Freud E, Macdonald SN, Chen J, Quinlan DJ, Goodale MA, Culham JC. 2018 Getting a grip on reality: grasping movements directed to real objects and images rely on dissociable neural representations. Cortex **98**, 34-48. (10.1016/j.cortex.2017.02.020) 28431740

[105] Ozana A, Ganel T. 2019 Weber's law in 2D and 3D grasping. Psychol. Res. **83**, 977-988. (10.1007/s00426-017-0913-3) 28871420

[106] Ozana A, Berman S, Ganel T. 2020 Grasping Weber's law in a virtual environment: the effect of haptic feedback. Front. Psychol. **11**, 1-15. (10.3389/fpsyg.2020.573352) 33329216 PMC7710620

[107] Rzepka AM, Hussey KJ, Maltz MV, Babin K, Wilcox LM, Culham JC. 2022 Familiar size affects perception differently in virtual reality and the real world. *Figshare*. (10.6084/m9.figshare.c.6251532) PMC9745877 36511414

